# Impact of Protoporphyrin Lysine Derivatives on the Ability of *Nosema ceranae* Spores to Infect Honeybees

**DOI:** 10.3390/insects11080504

**Published:** 2020-08-05

**Authors:** Katarzyna Buczek, Kamil Deryło, Mateusz Kutyła, Katarzyna Rybicka-Jasińska, Dorota Gryko, Grzegorz Borsuk, Beata Rodzik, Mariusz Trytek

**Affiliations:** 1 Department of Industrial and Environmental Microbiology, Faculty of Biology and Biotechnology, Maria Curie-Skłodowska University, Akademicka 19, 20-033 Lublin, Poland; romanczuk.kat@gmail.com (K.B.); mateusz.kutyla@poczta.umcs.lublin.pl (M.K.); 2 Department of Molecular Biology, Faculty of Biology and Biotechnology, Maria Curie-Skłodowska University, Akademicka 19, 20-033 Lublin, Poland; kamil@hektor.umcs.lublin.pl; 3 Institute of Organic Chemistry, Polish Academy of Sciences, Kasprzaka 44/52, 01-224 Warsaw, Poland; katarzyna.rybickajasinska@gmail.com (K.R.-J.); dgryko@gmail.com (D.G.); 4 Institute of Biological Basis of Animal Production, Faculty of Biology, Animal Sciences and Bioeconomy, University of Life Sciences in Lublin, Akademicka 13, 20-950 Lublin, Poland; grzegorz.borsuk@up.lublin.pl; 5 Department of Applied Mathematics, Faculty of Mathematics, Maria Curie-Skłodowska University, Plac Marii Curie-Skłodowskiej 1, 20-031 Lublin, Poland; beata.rodzik@poczta.umcs.lublin.pl

**Keywords:** infected honeybees, microsporidia, *Nosema ceranae*, feed intake, protoporphyrin IX amides

## Abstract

**Simple Summary:**

Honeybees, which are important for the development and maintenance of natural ecosystems, are infected by microsporidia, *Nosema apis* and *N. ceranae*. These parasites induce a disease named nosemosis contributing to the impairment of digestion and nutrient absorption, ultimately leading to total colony collapse. The need for research into the control of *N. ceranae* has become increasingly important. Promising compounds for the treatment of nosemosis are porphyrins. In the present study, we examined the effects of three different porphyrins on the infectivity of *N. ceranae* microsporidia. A significantly lower level of infection was observed in the bees infected with the porphyrin-treated spores than in the control bees (infected with untreated spores). We showed that protoporphyrin lysine derivatives in particular prevented the development of *Nosema* spores and simultaneously extended bee life spans (up to 50%). The results also indicate that these porphyrins may contribute to the reduction in digestive nutrient absorption disorders in bees. The present findings can be used to develop a new class of drugs for combating nosemosis. These compounds may serve as preventive or disinfection agents through direct inactivation of *Nosema* both in the midgut and outside the host body, i.e., in the hive.

**Abstract:**

The effect of two protoporphyrin IX derivatives conjugated with single (PP[Lys(TFA)-OH)]_2_) or double (PP[Lys(TFA)-Lys(TFA)-OH]_2_) lysine moieties on the infectious capacity of *Nosema ceranae* spores was examined, and their efficacies were compared with those of a cationic porphyrin (H_2_TTMePP). Honeybees were inoculated with spores preincubated with porphyrins or with untreated spores (control). A significantly lower level of infection was observed in the bees infected with the porphyrin-treated spores than in the infected control. Porphyrins 1 and 2 reduced the infectious capability of microsporidia more efficiently than porphyrin 3, with bee mortality declining to almost 50%. Confocal analysis of the midguts of infected bees revealed distinct differences in the number of spores between the control group and the group infected with PP[Lys(TFA)-Lys(TFA)-OH]_2_-treated spores. Notably, bees with a reduced level of infection consumed less sucrose syrup than the control bees, indicating a reduction in digestive disorders and an improvement in food absorption.

## 1. Introduction

*Nosema ceranae* is a parasite and member of the Microsporidia division, which is a group of obligate intracellular parasites that can infect vertebrate and invertebrate species [1,2]. *Microsporidia* are fungi [3,4,5,6,7] that exist outside the host cell only as metabolically inactive spores [8].

Honeybees, which are important for the development and maintenance of natural ecosystems, are infected by two species of microsporidia, *Nosema apis* and *N. ceranae*, and they both cause the disease nosemosis [9,10,11,12,13]. However, in North America and Europe, infections caused by *N. ceranae* have become increasingly common, and *N. ceranae* exhibits stronger virulence than *N. apis* [14,15,16,17]. Exposure of honeybees to xenobiotic pesticides and other environmental chemicals increases their susceptibility to *N. ceranae* infection upon exposure to the parasite [18,19]. The synergic interaction between chemicals and *Nosema* is reflected in the low survival rate of honeybees exposed to both of these factors [20]. Infection of the host by *Nosema* occurs after the ingestion of spores with food or water [21,22]. *N. ceranae* can be spread via trophallaxis (food exchange), which can potentially increase colony infection [23,24]. However, although they are eager to ingest food, infected bees are less likely to share it with other bees, which may indicate a higher level of hunger in these bees [25]. The source of disease may also be combs contaminated with bee feces. In a very recent report, *Nosema* spores were also shown to be transferred by air in an apiary [26]. Spores develop in the midgut of honeybees, followed by the polar tube extrusion and injection of the sporoplasm inside epithelial cells. This results in serious health problems characterized by immune suppression [27,28], the degeneration of intestinal epithelial cells [2], the impairment of digestion and nutrient absorption and shortened honeybee life spans [21,29,30]. *N. ceranae* infections cause increased energetic stress in bees [29]. These adverse outcomes ultimately contribute to total colony collapse in Europe and North America [31,32,33,34,35].

The only effective treatment for the *Nosema* microsporidial infections is the antibiotic fumagillin [32,36], isolated from the fungus *Aspergillus fumigatus* [37]. Fumagillin is currently banned in Europe because it causes severe toxic side effects in human subjects, and furthermore, it has no established maximum residue limit (MRL) in honeybee products [38,39,40]. Moreover, *N*. *ceranae* has been found to become resistant to this antibiotic over time [39]. Due to the lack of an effective nosemosis suppressant, various compounds, e.g., essential oils and plant extracts, have been subjected to intensive research [40]. Additionally, propolis, produced from resinous substances collected from plants and used by bees to protect their nests from parasites and pathogens, is studied as an alternative to fumagillin [41,42]. Promising compounds for the treatment of nosemosis are porphyrins [43]. Porphyrins are organic heterocyclic compounds consisting of four pyrrole rings connected to each other by methine bridges. In a wide variety of organisms, from aerobic microbes to humans, the iron protoporphyrin IX (PPIX) complex plays a crucial role in a number of proteins, where it binds to polypeptide chains, e.g., hemoglobin, myoglobin, cytochrome c, peroxidase, and catalase. However, due to the hydrophobic characteristic of porphyrins, different hydrophilic groups (e.g., amino acids) are conjugated to the porphyrin periphery, which extends their applicability in medical purposes [44]. Porphyrins have already been shown to be effective in vitro in the photodynamic inactivation of bacteria, viruses, fungi, and protozoa [45,46]. The mode of action of this inactivation involves the formation of reactive oxygen species (ROS) [47,48,49]. They are also used as anticancer drugs based on photosensitization mechanisms [50,51].

In a previous work, protoporphyrin conjugated to aspartate moieties was shown to reduce the ability of *N. ceranae* spores to develop in honeybees [43]. The aim of the present study was to determine the infectious capability of this microsporidian parasite after pre-treatment with two different PPIX derivatives bearing lysine moieties that differ in chain length. Experiments were carried out in vivo with caged honeybees that were infected with porphyrin-treated spores, and the results were compared to those achieved by treatment with the cationic porphyrin H_2_TTMePP.

## 2. Materials and Methods

### 2.1. Chemicals

**Figure 1:**
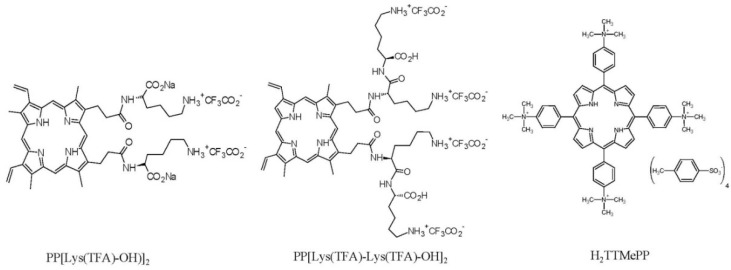
Molecular structures of the porphyrins used in the study.

The PPIX amides with amino acid moieties as hydrophilic head groups (Figure 1) were obtained from the Institute of Organic Chemistry PAN in Warsaw. PP[Lys(TFA)-OH)]_2_ was synthesized from PPIX, as described by Maximova et al. [52]. The porphyrin with two lysine moieties, PP[Lys(TFA)-Lys(TFA)-OH]_2_, was synthesized using a solid-phase technique and fully characterized (Figure S1). The compound 5,10,15,20-tetrakis[4 -(trimethyl-ammonio)phenyl]-21H,23H-porphine tetra-p-tosylate (H_2_TTMePP) (Figure 1) was purchased from Sigma-Aldrich (Saint Louis, MO, USA) 

### 2.2. Pre-Treatment of Microsporidia with Porphyrins (In Vitro Study)

The *Nosema* spores for the study were isolated from *Nosema*-infected honeybees (*Apis mellifera carnica*) collected from the experimental apiary of the University of Life Sciences in Lublin. The *Nosema* species was identified using a PCR-based test [31,53]. The presence of *N. ceranae* DNA was demonstrated by the detection of the specific 16S rDNA (Figure S2).

First, intestines prepared from 50 honeybees were gently homogenized on ice in 50 mL of sterile water; a fresh suspension of spores was centrifuged (13,000 rpm; 30 min) and washed twice with H_2_O (2000 rpm/5 min) and once with sterile phosphate-buffered saline (PBS). Then, the concentration of the spores was adjusted to 2 × 10^7^ mL in 0.5% sucrose solution. Subsequently, the spore suspension was divided into four portions. The first spore portion was suspended in 0.5% sucrose solution containing PP[Lys(TFA)-OH)]_2_ (100 μM); the second portion was suspended in a sucrose solution containing PP[Lys(TFA)-Lys(TFA)-OH]_2_ (100 μM); the third portion was suspended in a sucrose solution containing H_2_TTMePP (100 μM), and the fourth portion was left untreated in sucrose solution as the control. All spore suspensions were incubated in the dark for 24 h at 30 °C, with gentle shaking using a Roto-Bot rotator (Benchmark Scientific, Sayreville, NJ, USA). Next, the spores were centrifuged (4000 rpm/15 min) and washed extensively using a sterile, aqueous 0.9% sodium chloride solution and then a 0.5% sucrose solution. The procedure was repeated four times to remove porphyrin residues. The control was subjected to the same pre-treatment procedure but with a spore suspension devoid of porphyrin. Prior to infection, the spore suspensions obtained were adjusted to have inocula with equal concentrations (3 × 10^7^ spores/mL) [54]. Each inoculum was freshly prepared on the day of infection by mixing with 50% sucrose solution.

### 2.3. Infection of Honeybees with Porphyrin-Pre-Treated Spores (In Vivo Study)

One day post-emergence, *Nosema*-free (confirmed by PCR) honeybees (*A. mellifera carnica*) were divided randomly into four groups with 5 cages each (40 bees per cage) and fed sugar syrup ad libitum. Three days post-emergence, the honeybees were starved for 2 h, anesthetized with CO_2_ and then placed inside Eppendorf tubes (0.5 mL) with the bottom cut off to facilitate individual feeding. Five microliters of 50% sucrose solution containing 150,000 *N. ceranae* spores that were pre-treated with porphyrins were administered to each bee of the three experimental groups using a micropipette. Honeybees in the control group were infected with porphyrin-untreated control spores. The CO_2_ anesthesia lasted a short time, i.e., until the disappearance of abdominal reflexes (ca. 1 min), to facilitate placing the individual bees in Eppendorf tubes. Bees that were not placed in the Eppendorf tubes or served as controls were euthanized in the same way to eliminate the effect of the anesthesia factor on longevity, as it is well known that CO_2_ shortens the life of bees [55]. After inoculation, bees were returned to their cages and fed ad libitum with 50% (w/v) sucrose solution throughout the remainder of the experiment. The cages were placed in a laboratory chamber with regulated temperature and humidity. On the 7th, 12th, and 20th day post-infection (p.i.), the level of infectivity of the pre-treated and untreated spores was determined by counting the microsporidia that developed in living honeybees. For this purpose, ten honeybees (two specimens from each of the five cages) were homogenized in 10 mL of sterile, distilled water, and the number of *N*. *ceranae* spores was counted according to the method described by Hornitzky [56] and Fries et al. [57]. Additionally, the number of dead bees was recorded throughout the experiment, and feed intake was evaluated daily by measurement of the decline in the food volumes in 5 mL syringes in the experimental groups (reading from marks on a graduated scale).

### 2.4. Visualization and Determination of the Abundance of Nosema Spores in the Intestines of Honeybees

For the visualization of *Nosema* infection, the intestines from 5 live bees of each of the examined groups were isolated at the end of the experiments. The intestines were placed onto microscope slides and cut lengthwise with a scalpel under a light microscope to visualize the internal surface. The honeybee midgut tissue preparations were treated with the chitin-binding fluorescent agent Calcofluor White M2R (CFW) (Sigma-Aldrich, Saint Louis, MO, USA), according to methods described by Gerphagnon et al. [58], Green et al. [59], and Snow [60], with some modifications. The *Nosema* spores in the honeybee intestine were identified and quantified by laser scanning confocal microscopy (LSM780, Zeiss, Jena, Germany). The microscopic analysis was carried out with a 405 nm laser for optimal excitation of the fluorophore and two PMT (PhotoMultiplier Tube) detectors operating in ranges corresponding to fluorescence emitted by CFW. The number of *Nosema* spores (ovals with a blue cell wall) was counted at a 405 nm excitation wavelength and a detection bandwidth (600–700 nm) used for viewing CFW staining. At least four different intestine regions (fields of view) were analyzed in each sample (Table S1).

### 2.5. Statistical Analysis

Statistical analysis was performed using Kruskal–Wallis ANOVA and multiple comparisons of mean ranks. Statistical significance was assumed at a *p*-value of < 0.05. The mortality was analyzed by creating Kaplan–Meier survival curves for the bees in each group. The curves were compared using a log-rank post hoc test to determine which curves were significantly different from one another.

Spearman’s rank correlation analysis was performed to estimate the relationship between the feed intake and the number of spores.

## 3. Results

### 3.1. Influence of Porphyrins on Reducing the Infective Capacity of Nosema ceranae Spores

**Figure 2:**
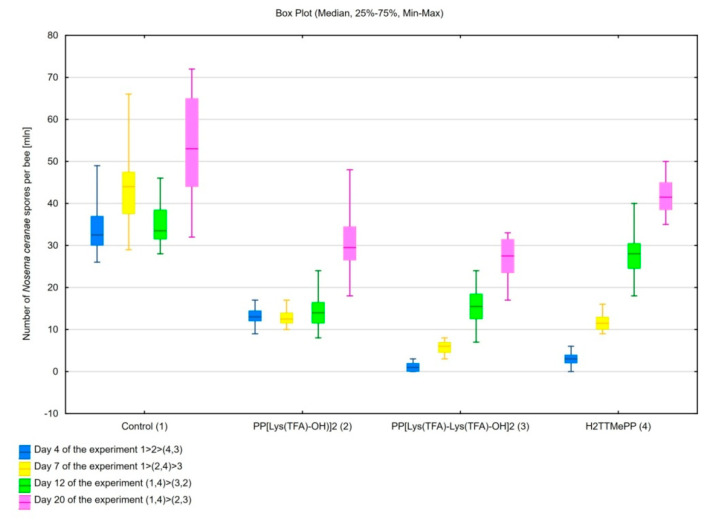
Impact of incubation of *Nosema ceranae* microsporidia with porphyrins on the number of spores in honeybees. The differences between the groups are shown in the figure legend (*p* < 0.01; Kruskal-Wallis test).

Honeybees infected with spores, incubated separately with each of the porphyrins—H_2_TTMePP, PP[Lys(TFA)-OH)]_2_, and PP[Lys(TFA)-Lys(TFA)-OH]_2_—for 24 h showed significantly decreased spore counts after day 7 compared to the honeybees in the control group, which were exposed to untreated microsporidia (H_(3, N = 80)_ = 67.75; *p* < 0.001). After days 12 (H_(3, N = 80)_ = 59.91; *p* < 0.001) and 20 (H_(3, N = 80)_ = 56.17; *p* < 0.001) of the experiment, the lowest number of spores was observed in bees infected with spores preincubated with porphyrins PP[Lys(TFA)-OH)]_2_ (*p* < 0.001) and PP[Lys(TFA)-Lys(TFA)-OH]_2_ (*p* < 0.001). Although H_2_TTMePP spores caused slightly lower spore loads in honeybees on days 12 and 20 than the control spores, differences between these groups were not significant. On the 12th and 20th day p.i., the number of spores in the PP[Lys(TFA)-OH)]_2_-treated group was 2.4- and 1.8-fold less, respectively, than that in the control group. The preincubation of spores treated with PP[Lys(TFA)-Lys(TFA)-OH]_2_ resulted in 2.2 and 2-fold decreases in the number of spores in honeybees (Figure 2).

**Figure 3:**
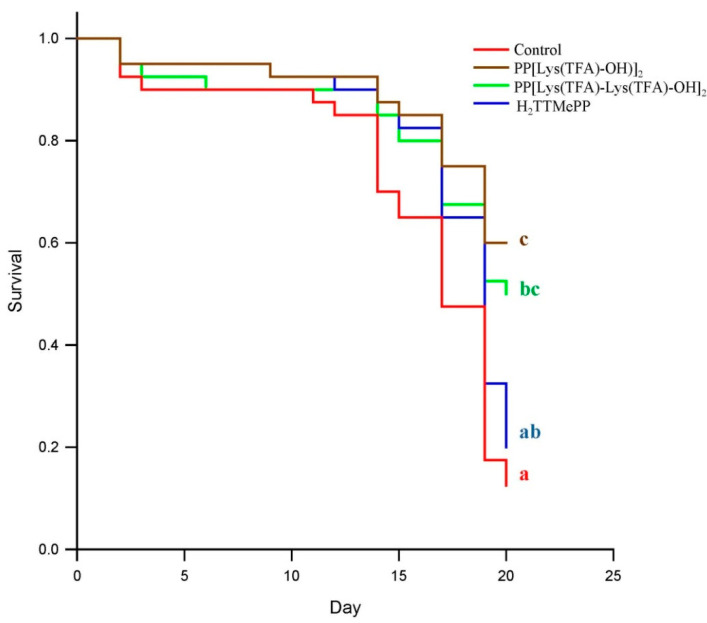
Kaplan-Meier survival curves for honeybees after infection with *Nosema ceranae* microsporidia treated with porphyrin solutions. A log-rank post hoc test was used to determine which curves were significantly different from each other (*p* = 0.001). Curves labeled with the same letter are not significantly different.

Moreover, the mortality rates of infected honeybees were significantly decreased within 20 d of infection. Lower mortality (39.5%, 48.1%, 79.5%) was observed in honeybees infected with PP[Lys(TFA)-OH)]_2_, PP[Lys(TFA)-Lys(TFA)-OH]_2_, and H_2_TTMePP-treated microsporidia than in the control group of bees (87.9%) (Figure 3). Statistically significant differences were observed on the 20th day of the experiment for the PP[Lys(TFA)-OH)]_2_ group in comparison with the control group (*F* = 3.08; *p* = 0.01).

### 3.2. Feed Intake by Honeybees

**Figure 4:**
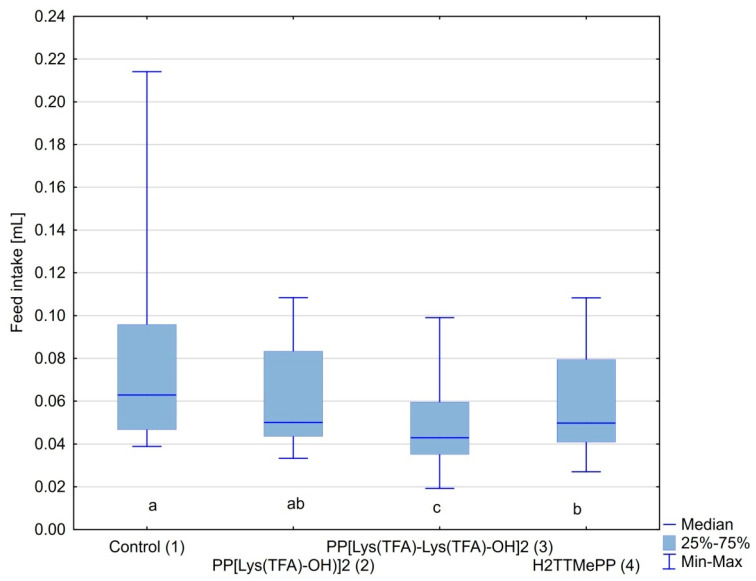
The amount of syrup consumed per bee per day. Lowercase letters (**a**–**c**) indicate the differences among the groups (*p* < 0.05; Kruskal-Wallis test).

The *Nosema-*infected bees in all experimental groups consumed the syrup willingly. The amount of syrup consumed by honeybees in the control cages was on average 0.09 mL per bee per day and was the highest. The amount of food consumed in groups treated with the infected porphyrin spores was 0.05 and 0.04 mL (per bee, per day) for PP[Lys(TFA)-OH)]_2_ and PP[Lys(TFA)-Lys(TFA)-OH]_2_, respectively (Figure 4). The feed intake was also lower in groups infected with H_2_TMePP-treated spores than in the control group. However, a significant reduction in feed intake was observed in bees infected with PP[Lys(TFA)-Lys(TFA)-OH]_2_ (*p* < 0.001) and H_2_TMePP (*p* = 0.006). The feed intake was correlated with the number of spores (Spearman *R* = 0.55; *p* < 0,001), i.e., the higher the number of spores, the higher the feed intake.

**Figure 5:**
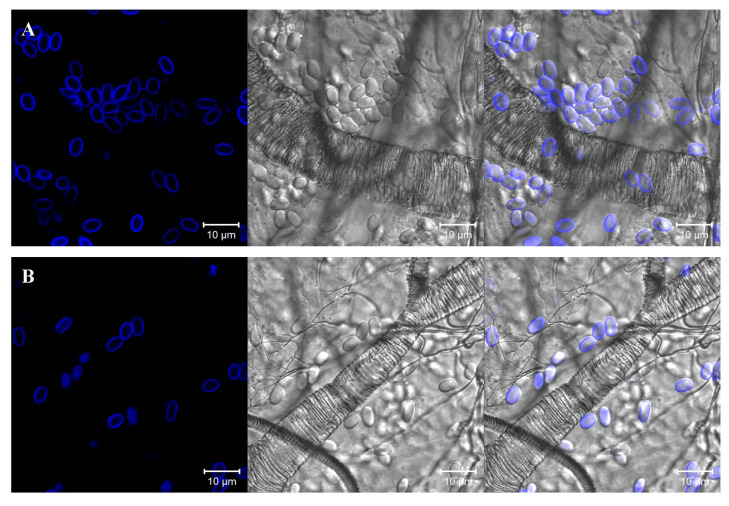
Confocal microscopic images of the midguts of honeybees infected with untreated (**A**) and porphyrin-treated (**B**) *Nosema* spores. *λ_exc_* = 405 nm.

A higher number of spores (ovals with cell wall emitting blue fluorescence) were observed in the midgut images of the control bees infected with untreated spores than in the midgut images of honeybees infected with PP[Lys(TFA)-Lys(TFA)-OH]_2_-treated spores (Figure 5).

## 4. Discussion

Microsporidia are parasites propagating exclusively in infected host cells. Studies have suggested that *N. ceranae* is more virulent to bees and bee colonies than *N. apis* [61,62,63]. The PCR amplification of the 16S rRNA gene of the spores that were isolated from honeybees (collected from infected colonies of the apiary of the University of Life Sciences in Lublin) resulted in PCR products (218–219 bp) corresponding to the DNA of *N. ceranae* spores (Figure S1).

With the detection of the parasite *N. ceranae* in European honeybees [10,21], the need for research into the control of this parasite has become increasingly important. Various compounds against *Nosema* spp., one of which is a PPIX amide derivative, have been investigated [40,43]. PPIX is used in biotechnology research and biomedicine, especially in photodynamic therapy (PDT) [51,64]. To our knowledge, there are few investigations of the effect of potential compounds on the infection capability of *N. ceranae*. In previous studies, PPIX derivatives conjugated to aspartate and dissolved in sucrose solutions showed biological activity against *N. ceranae* microsporidia [43]. In the present study, we examined the effects of the two protoporphyrin lysine derivatives and, comparatively, a commercial cationic porphyrin on the infectivity of *Nosema* microsporidia. Spores isolated from honeybees were preincubated with the porphyrins that were used to infect honeybees in in vivo tests. A higher *N. ceranae* infection rate was observed in the honeybee control group infected with untreated spores. The high level of infection (on average over 42 million spores per bee) was maintained throughout the experiment in this group. The statistically insignificant decrease in the number of spores observed on day 12 compared to day 7 may be related to temporary activation of natural immune pathways in the bee organism. The number of spores in bees infected with preincubated spores significantly decreased compared to that in the control bees (Figure 2). The propagation of *Nosema* spores in infected honeybees was most likely prevented by the inactivation of the *Nosema* spores with the porphyrins. These compounds were used at a 100 µM concentration, since the porphyrin PP(Asp)_2_ had a limiting effect on the infectious capacity of spores at this concentration [43]. To our knowledge, there are limited in vitro investigations of the anti-*Nosema* activity of compounds. In a study by Gisder and Genersch, who used IPL-LD 65Y cell cultures for *Nosema* cell propagation, two of ten tested compounds (clioquinol and metronidazole) proved to be very effective against *Nosema* spores, as determined by RT-PCR-ELISA [65]. However, these compounds acted at a much higher concentration (in the range of 300 µM to 1.2 mM) than the porphyrins used in the present study. In addition, these compounds showed cytotoxicity to IPL-LD 65Y cells [65]. Porphyrin compounds, in contrast, have not induced any adverse effects in our studies so far [43,66], showing that they could be effective against nosemosis without causing harm to the host. Our recent studies [66] confirmed that the protoporphyrin lysine derivatives are not toxic to honeybees in contrast to the zinc-coordinated porphyrin. The latter, despite its high activity in the reduction of the spore numbers (both in vitro and in vivo), also increased the mortality of honeybees within 23 d post infection. Numerous natural compounds are widely tested on live bees for their anti-*Nosema* potential. Borges and colleagues have examined 12 compounds, of which only hydroxytyrosol and trans-cinnamaldehyde did not show a significant effect on *Nosema* spore counts. They showed high potential of nutraceutical and immuno-stimulatory compounds to control *N. ceranae* to varying extents, but, as in the case of porphyrins, this issue requires further in-depth research [67]. There are also investigations of the intestinal microbiome of bees, which contributes substantially to improvements in bee immunity but does not directly control *Nosema* spp. [68,69].

In the present study, both PPIX derivatives induced a similar effect on the ability of spores to infect bees, with a slight predominance of PP[Lys(TFA)-Lys(TFA)-OH]_2_ with double amino acid moieties. H_2_TTMePP caused no substantial decrease in the infectivity of microsporidia. This indicates that the amphiphilic porphyrins bearing peptides with lysine moieties exert higher activity than the cationic porphyrins. Porphyrins conjugated with peptides have been reported to exert enhanced bioactivity against microorganisms [70,71]. The results showed that PP[Lys(TFA)-Lys(TFA)-OH]_2_ in particular prevented the development of spores and simultaneously extended bee life spans (Figure 3). Mortality was significantly (up to 50%) lower in the bees that had ingested microsporidia treated with porphyrin that contained lysine moieties than in the bees from the control cages and was also lower than that in the bees infected with spores treated with H_2_TTMePP. These results are consistent with our previous study, which showed that PP(Asp)_2_ was more effective than another cationic porphyrin, TTMePyP, in preventing the development of *Nosema* in honeybees [43]. The differences in the levels of infection have been shown by CFW tissue staining. This fluorescent blue dye binds to β1–4 polysaccharides, as it did in the case of microsporidia in the isolated bee intestines. Confocal microscopic images of the midguts of honeybees infected with porphyrin-treated and untreated spores showed distinct differences in the number of spores. The highest number of spores was observed in the intestines of bees from the control *Nosema*-infected group (Figure 5). We also observed that the amount of food consumed in this group was accordingly 1.5 and 2-fold higher than that of the porphyrin-treated groups. This may indicate that a higher level of infection increases the need for food in bees [25], which can be explained by the fact that *Nosema ceranae* spore infection enhances sugar metabolism in honeybees [22]. Moreover, *Nosema* microsporidia contribute to the degeneration of epithelial cells in the midgut [21], causing a decline in the digestion and absorption of food and leading to increased hunger levels and demands for nutrients in bees. Therefore, porphyrins may contribute to the reduction in digestive nutrient absorption disorders in bees.

## 5. Conclusions

Our study showed a distinct decrease in the infectious capacity of *Nosema ceranae* spores as a result of treatment with protoporphyrin lysine derivatives. *N. ceranae* spores exposed to these porphyrins underwent limited development in honeybees, resulting in a reduction in mortality rates. In addition, a decrease in the level of *Nosema* infection has been associated with a reduced nutritional requirement in honeybees. Our findings indicate that PPIX derivatives with expanded lysine moieties can be beneficial for controlling *N. ceranae* infections. Since the results of the study confirmed their direct impact on the spores, these compounds may also serve as preventive or disinfection agents through direct inactivation of *Nosema* both in the midgut and outside the host body, i.e., in the hive. However, the mechanism underlying the activities of porphyrins against microsporidia is still unclear and requires further in-depth research, and other variations in the modification of PPIX-bearing lysine moieties are needed to obtain compounds with the ability to completely inactivate *Nosema* spores, thus limiting propagation in host cells.

## Supplementary Materials

The following are available online at https://www.mdpi.com/2075-4450/11/8/504/s1. Table S1: Number of *Nosema* spores in honeybee intestines. The quantification analysis was performed using confocal microscopic images after CFW intestinal staining, Figure S1: Spectra of the PP[Lys(TFA)-Lys(TFA)-OH]_2_, Figure S2: Agarose gels (2%) showing PCR products amplified from *Nosema ceranae* DNA extracted from spores isolated from *Nosema*-infected honeybees collected from the experimental apiary of the University of Life Sciences in Lublin.
