# Supplementary material for: Impact of Protoporphyrin Lysine Derivatives on the Ability of Nosema ceranae Spores to Infect Honeybees
Source: Insects. 2020 Aug 5;11(8):504. doi: 10.3390/insects11080504 (PMC7469180; doi:10.3390/insects11080504)
Supplement: Supplementary file 1 [file insects-11-00504-s001.pdf]

Supplementary Materials

Table S1. Number of *Nosema* spores in honeybee intestines. The quantification analysis was performed using confocal microscopic images after CFW intestinal staining.

Group	Average Number of *Nosema* Spores in Isolated Intestines
Control	55.2
PP[Lys(TFA)-OH)]$_2$	30.36
PP[Lys(TFA)-Lys(TFA)-OH]$_2$	27.95
H_2TTMePP	44.4

Synthesis

PP[Lys(TFA)-Lys(TFA)-OH]$_2$ was synthesized manually by Fmoc chemistry on a 0.02-mmol scale. Fmoc-Lys(Boc) (4 equiv.) was attached to Wang resin (Fmoc-Lys-Wang resin), and PP(IX) was subsequently attached to the Fmoc-Lys(Boc)-Glu-Wang resign. Fmoc deprotection was performed with 20% piperidine in DMF (1.5 mL, 1-2 h), and coupling was achieved using HBTU (6 equiv.) and DIPEA (6 equiv.) in DMF (2 mL). After the final coupling, the resin was washed with DMF (5 x 1 mL) and DCM (5 x 1 mL) and dried. The product was cleaved from the resin with TFA/DCM (25%, v/v) with a catalytic amount of anisole in 3 h. The obtained crude product (PP[Lys(TFA)-Lys(TFA)-OH]$_2$) was precipitated with Et_2O, centrifuged and subsequently purified by RP column chromatography (eluent: H_2O:MeOH:TFA 95:94.5:0.5 (v/v/v) mixture).

^{1}H NMR (500 MHz, TFA-d_1): = 10.41–10.24 (m, 4H, meso), 8.59–8.49 [m, 2H, -CH = (vinyl)], 8.17–8.10 [m, 4H, -CH = (vinyl), = CH-], 7.82–7.55 (m, 16H, -CH-, -CH_2), 6.48 (dd, J = 19.7, 6.7 Hz, 2H, -CH_2), 6.25 (d, J = 11.5 Hz, 2H, -CH_2), 4.39–4.26 (m, 5H, -CH-, -CH_2), 4.12–4.05 (m, 2H, -CH-), 3.76 (d, J = 9.25 Hz, 6H, por-CH_3), 3.64 (d, J = 12.9 Hz, 6H, por-CH_3), 3.56–3.42 (m, 2H, -CH-), 3.21–3.10 (m, 4H, -CH-, -CH_2), 2.81–2.69 (m, 6H, -CH-, -CH_2), 2.40–2.31 (m, 3H, -CH-), 1.69–1.61 (m, 2H, -CH-), 1.59–1.41 (m, 5H, -CH-, -CH_2), 1.39–1.28 (m, 5H, -CH-, CH_2), 1.23–1.14 (m, 2H, -CH-), 1.06–0.96 (m, 2H, -CH-), -3.78 (br s, 2H, NH) ppm.

^{13}C NMR (125 MHz, DMSO-d_6): = 173.3, 171.7, 158.1, 158.1, 157.9, 157.8, 130.0, 130.0, 121.3, 118.3, 115.9, 115.9, 113.4, 112.6, 54.5, 52.1, 51.6, 40.1, 40.0, 39.9, 39.8, 39.8, 39.7, 38.5, 38.3, 38.1, 31.4, 30.2, 30.2, 26.5, 26.4, 22.3, 21.9, 21.8, 12.6, 12.6, 11.4 ppm.

HRMS-ESI: m/z $[M + H]^+$ calcd for $C_{58}H_{83}O_8N_{12}$: 1075.6457; found: 1075.6451.

UV/Vis (MeOH): $_{max}$ ( ) = 401 (9.40 x 10^4), 504 (7.94 x 10^3), 535 (6.44 x 10^3), 576 (4.11 x 10^3), 627 (2.73 x 10^3).

^{1}H NMR and ^{13}C NMR SPECTRA of the Porphyrins; PP[Lys(TFA)-Lys(TFA)]$_2$

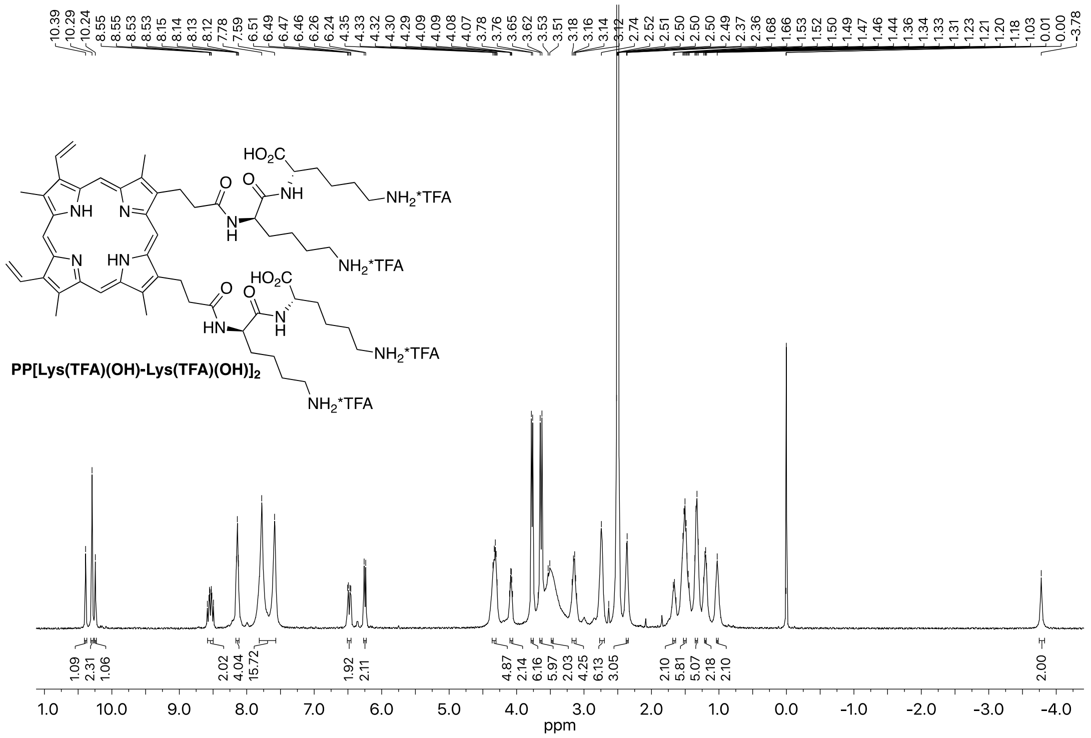

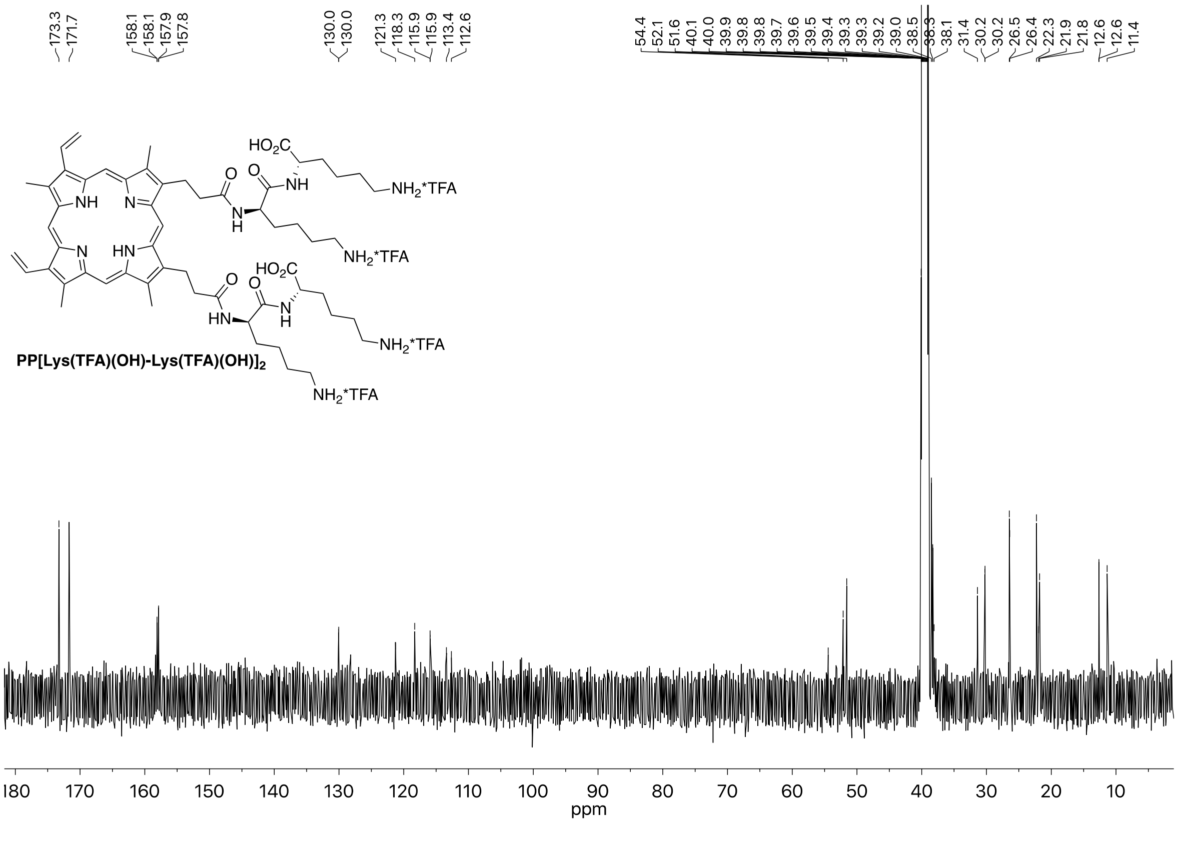

Figure S1. Spectra of the PP[Lys(TFA)-Lys(TFA)-OH]2.

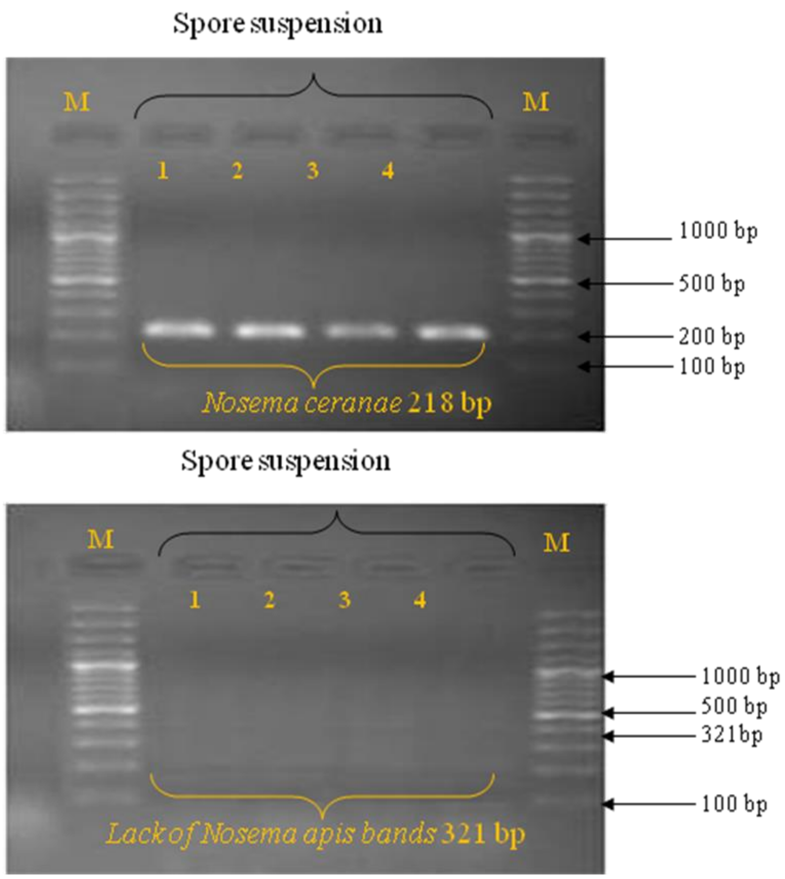

Figure S2. Agarose gels (2%) showing PCR products amplified from Nosema ceranae DNA extracted from spores isolated from Nosema-infected honeybees collected from the experimental apiary of the University of Life Sciences in Lublin.
